# Omalizumab Therapy for Bullous Pemphigoid: A Case Report

**DOI:** 10.7759/cureus.100410

**Published:** 2025-12-30

**Authors:** Adel Alsantali, Reema S AlZaidi, Bader Alharbi, Ahmed K Bakhsh, Anhar K Zahrani, Mohammed I Alturkistani

**Affiliations:** 1 Dermatology, King Fahad Armed Forces Hospital, Jeddah, SAU; 2 College of Medicine, Umm Al-Qura University, Makkah, SAU

**Keywords:** autoimmune blistering disease, bullous pemphigoid, ige-targeted therapy, omalizumab, steroid resistance

## Abstract

Bullous pemphigoid (BP) is a chronic autoimmune blistering disease characterized by the presence of autoantibodies that target hemidesmosomal proteins, specifically BP180 and BP230. This immune response leads to the formation of subepidermal blisters and inflammation. The primary treatment for BP involves systemic corticosteroids; however, long-term use can result in significant adverse effects, and some patients may experience resistance to steroid therapy. We report the case of a 62-year-old male with multiple comorbidities who developed progressive, itchy blisters and extensive skin erosions affecting more than 50% of his body surface. Although he initially showed improvement with intravenous corticosteroids, new blister formation continued, and Staphylococcus aureus bacteremia prevented further use of immunosuppressive therapy. The patient was managed with targeted antibiotics, intravenous immunoglobulin (IVIG), and omalizumab. This treatment resulted in significant clinical improvement, the resolution of new blister formation, and a successful tapering of corticosteroids. Omalizumab has demonstrated promising efficacy in treating BP, especially in refractory cases. Given its favorable safety profile, further clinical trials are needed to determine its long-term role in the management of BP.

## Introduction

Bullous pemphigoid (BP) is a chronic autoimmune blistering skin disease caused by autoantibodies targeting hemidesmosomal proteins (BP180 and BP230) in the basement membrane, leading to subepidermal blister formation and inflammation. It primarily affects the elderly, with a global incidence ranging from 0.21 to 7.63 per 100,000 people [1]. The European rates are between 2.5 and 42.8 per million annually. Its prevalence is also variable, reported between 1.46 and 47.99 per 100,000 people [2], with a meta-analysis estimating a global cumulative incidence of 8.2 per million, higher in Europe 10.3 per million, compared to Asia 5.6 per million [2]. Systemic corticosteroids remain the standard treatment, but their long-term use is associated with serious adverse effects, including osteoporosis, diabetes, hypertension, and increased infection risk. Despite being the standard treatment, steroid resistance occurs in a subset of patients, as demonstrated in randomized trials where BP cases remained uncontrolled despite high-dose corticosteroids [3]. As a steroid-sparing alternative, omalizumab, a monoclonal antibody targeting IgE, has shown promising efficacy by neutralizing IgE-mediated inflammatory pathways involved in BP pathogenesis. Studies report complete remission rates of approximately 67.9% to 84% in patients treated with omalizumab, with significant improvement in pruritus and blister formation [4-6]. Additionally, omalizumab has been effective in refractory cases, including those unresponsive to systemic corticosteroids or rituximab [4]. Given its favorable efficacy and safety profile, omalizumab represents a viable therapeutic option, particularly for severe or treatment-resistant BP. Here, we present a case of BP successfully managed with omalizumab, contributing to the growing evidence supporting IgE-targeted therapy and emphasizing the need for further clinical trials to establish long-term efficacy and safety.

## Case presentation

**Figure 1:**
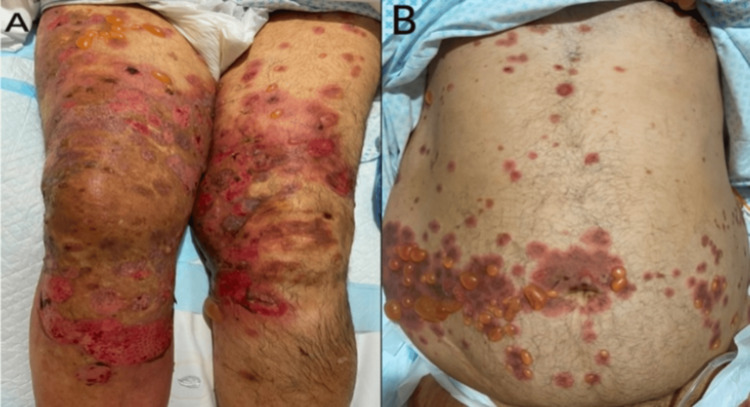
Multiple tense vesicles and bullae vary in size and arise within urticarial plaques. Some rupture, leaving erosions and hemorrhagic crusts over the lower extremities and trunk (A, B).

A 62-year-old male with a medical history significant for hypertension, bronchial asthma, chronic kidney disease, and pre-diabetes presented to the emergency department with a 20-day history of progressively worsening pruritic skin lesions and blistering involving the body. The patient also reported reduced oral intake, mild dysphagia, and dizziness. He denied any prior similar episodes. There was no history of fever, chills, recent infections, insect bites, or recent initiation of new medications. Further history revealed a positive family history of bullous pemphigoid, as his mother had been previously affected. The patient had been receiving long-term furosemide and perindopril. On physical examination, the patient was afebrile, with a blood pressure of 90/60 mmHg. Cutaneous examination revealed multiple generalized tense bullae arising on an erythematous, urticarial base, along with annular, arciform, and polycyclic urticarial plaques over the trunk. Numerous erosions and areas of skin detachment were observed, predominantly involving the lower extremities, with more than 50% body surface area involvement (Figure 1). Both Nikolsky and Asboe-Hansen signs were negative. No oral mucosal lesions were identified; however, solitary mild genital erosions were present. The right lower extremity was warm and edematous. Laboratory evaluation demonstrated leukocytosis (14.8 × 10^9^/L) with eosinophilia (1.74 × 10^9^/L). Inflammatory markers were elevated, with a C-reactive protein level of 29 mg/L. Serum immunoglobulin E levels were increased (498 kIU/L). Hepatitis serology and HIV testing were negative. Laboratory findings are summarized in Table 1, with abnormal values highlighted. Skin punch biopsies were obtained for hematoxylin-eosin staining and direct immunofluorescence (DIF). Histopathological examination revealed subepidermal blister formation with a dermal infiltrate composed of scattered perivascular and interstitial mixed inflammatory cells, including eosinophils. Direct immunofluorescence performed on perilesional skin demonstrated strong, diffuse linear deposition of C3 along the basement membrane zone, with focal, moderate linear IgG deposition. IgA staining was negative. These findings supported the diagnosis of bullous pemphigoid.

**Table 1:** Laboratory parameters for the patient. ALT - alanine aminotransferase; AST - aspartate aminotransferase; INR - International normalized ratio.

Parameters	Normal range (units)	Patient results
Hemoglobin	135-175 g/L	145 g/L
White blood count	3.3-10.8 × 10⁹/L	14.8 × 10⁹/L
Neutrophils	2-7.5 × 10⁹/L	9.04 × 10⁹/L
Lymphocytes	1.5-4 × 10⁹/L	1.89 × 10⁹/L
Eosinophils	0-0.4 × 10⁹/L	1.74 × 10⁹/L
Monocytes	0.2-0.8 × 10⁹/L	2 × 10⁹/L
Basophils	0-0.2 × 10⁹/L	0.009 × 10⁹/L
Platelets	150-450 × 10⁹/L	344 × 10⁹/L
Urea	2.8-7.3 mmol/L	9.8 mmol/L
Creatinine	64-111 µmol/L	101 µmol/L
Sodium	135-145 mmol/L	118 mmol/L
Potassium	3.5-5.1 mmol/L	5.28 mmol/L
Chloride	98-107 mmol/L	93 mmol/L
Bicarbonate	22-29 mmol/L	18 mmol/L
ALT	7-55 U/L	10 U/L
AST	8-48 U/L	13 U/L
Albumin	37-50 g/L	29 g/L
Prothrombin Time	12.6-14.61 seconds	15.9 seconds
INR	0.9-1.15	1.16
C-reactive protein	0-5 mg/L	29 mg/L
Immunoglobulin E (IgE)	0-100 kIU/L	498 kIU/L
Immunoglobulin A (IgA)	0.63-4.84 g/L	1.4 g/L

**Figure 2:**
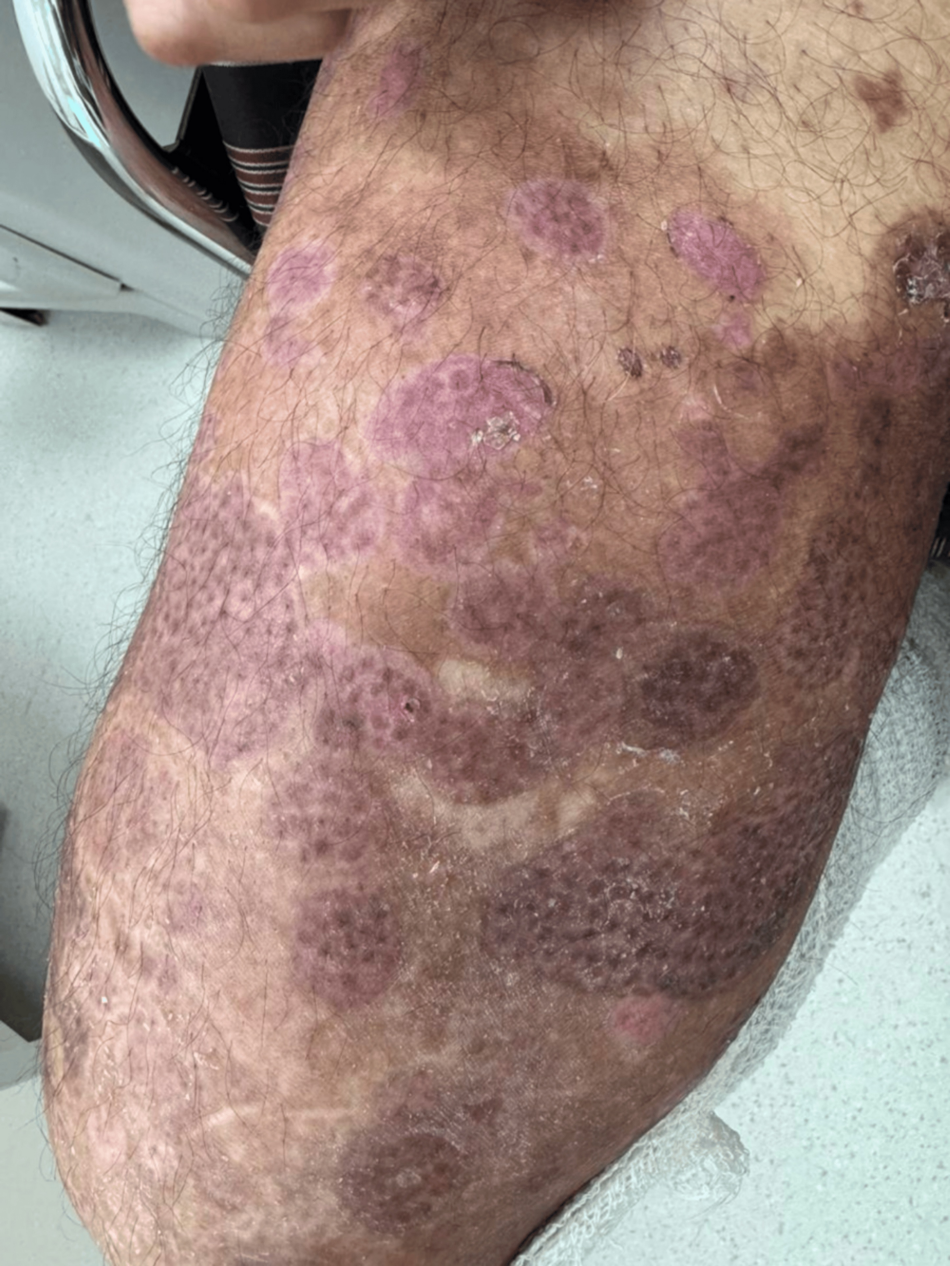
Full re-epithelization with post-inflammatory hyperpigmentation of the right thigh.

The patient was admitted for further evaluation and management. Initial treatment included a single dose of intravenous methylprednisolone (100 mg) and high-potency topical corticosteroids. Empiric intravenous piperacillin-tazobactam (4.5 g every six hours) and vancomycin (2 g every 12 hours) were initiated after blood cultures were obtained due to concern for bacteremia. Multidisciplinary consultations were obtained, including endocrinology, internal medicine, intensive care, gastroenterology, otolaryngology, and general surgery, to optimize systemic management and evaluate for potential complications or alternative sources of infection. Blood cultures subsequently grew gram-positive cocci, and systemic corticosteroids were withheld. Intravenous immunoglobulin (IVIG) was initiated at a total dose of 2 g/kg, administered over three days. Prior to the third dose, the patient developed bilateral lower limb pain and edema, accompanied by elevated D-dimer levels. Doppler ultrasonography was negative, and deep vein thrombosis was excluded; IVIG therapy was therefore resumed. The patient’s pruritus improved; however, blister formation persisted. After 72 hours, admission blood cultures grew methicillin-sensitive Staphylococcus aureus (MSSA). Broad-spectrum antibiotics were discontinued, and antimicrobial therapy was narrowed to flucloxacillin (2 g every four hours) and doxycycline (100 mg once daily). Omalizumab was initiated at a dose of 300 mg subcutaneously for two consecutive days, followed by 300 mg every two weeks. Oral prednisolone was subsequently started at 0.5 mg/kg/day and later increased to 0.75 mg/kg/day due to continued blister formation. During hospitalization, repeat blood cultures grew Pseudomonas aeruginosa, and ciprofloxacin (500 mg twice daily) was initiated. Fifteen days after admission, the patient demonstrated significant clinical improvement, with no new blister formation. By day 16, complete re-epithelialization of the affected skin was achieved (Figure 2). The patient was discharged on oral prednisolone at a dose of 0.7 mg/kg/day, with a taper of 5 mg every five days, along with completion of ciprofloxacin therapy and close outpatient follow-up.

## Discussion

Omalizumab works by binding to IgE, preventing its interaction with immune cell receptors and thereby reducing the inflammatory response associated with bullous pemphigoid, a process particularly relevant given the contribution of IgE autoantibodies to disease pathogenesis [7-10]. In our case, a 62-year-old male with hypertension, bronchial asthma, chronic kidney disease, and pre-diabetes presented with a 20-day history of progressive, pruritic blisters and extensive skin erosions involving over 50% of the body surface area. Although initial intravenous corticosteroids resulted in improvement of pruritus, the development of new blisters in the setting of Staphylococcus aureus bacteremia limited the use of further immunosuppressive therapy. The patient was therefore managed with targeted antibiotic therapy, intravenous immunoglobulin, and omalizumab. Following this approach, stabilization of disease activity was observed, with no further progression of lesions and clinical improvement prior to discharge, allowing a transition to a tapering regimen of oral prednisolone with scheduled omalizumab injections under close outpatient follow-up. Several case reports and small observational studies have described the use of omalizumab in patients with bullous pemphigoid, particularly in individuals with disease refractory to conventional therapies or in whom immunosuppressive agents are contraindicated [11-14]. In these reports, improvement in pruritus and blister formation has been observed after initiation of omalizumab, although the time to disease control and remission has varied, and treatment responses have been heterogeneous across studies [11-14]. Omalizumab has been administered either as monotherapy or as an adjunct to systemic therapies, including corticosteroids, in selected cases. While some studies have reported high response rates in corticosteroid-refractory patients [4], the available evidence remains limited by retrospective designs, small sample sizes, and lack of randomized controlled trials. Omalizumab has generally been reported as well-tolerated in published case reports and small series; however, hypersensitivity reactions, including anaphylaxis, have been reported in other approved indications [11,15]. The limitations of this report include its single-case design and the confounding impact of concurrent bacteremia, which restricted the use of additional immunosuppressive therapies.

## Conclusions

This case report suggests omalizumab as a good alternative therapy for BP. Furthermore, the role of omalizumab in BP management remains under investigation, and future studies are warranted to establish optimal dosing, treatment duration, and long-term outcomes. Given the promising response seen in steroid-resistant BP, ongoing research should explore whether omalizumab should be considered earlier in the treatment algorithm rather than being reserved for refractory cases. Future clinical trials may also help identify specific subgroups of BP patients who would benefit most from omalizumab, particularly those with predominant IgE-mediated disease. Additionally, understanding its long-term efficacy and relapse rates will be crucial in determining its place in BP management. While preliminary data suggest a steroid-sparing effect, more evidence is needed to define its potential as a first-line or maintenance therapy.
